# Influence of Disease Modifying Treatment, Severe Acute Respiratory Syndrome Coronavirus 2 Variants and Vaccination on Coronavirus Disease 2019 Risk and Outcome in Multiple Sclerosis and Neuromyelitis Optica

**DOI:** 10.3390/jcm12175551

**Published:** 2023-08-25

**Authors:** Jennifer Jessica Eisler, Giulio Disanto, Rosaria Sacco, Chiara Zecca, Claudio Gobbi

**Affiliations:** 1 Faculty of Biomedical Sciences, Università della Svizzera Italiana, 6900 Lugano, Switzerland; jennifer.jessica.eisler@usi.ch (J.J.E.); chiara.zecca@eoc.ch (C.Z.); 2 Department of Neurology, Neurocenter of Southern Switzerland (NSI), Regional Hospital of Lugano, Ente Ospedaliero Cantonale, 6900 Lugano, Switzerland; giulio.disanto@eoc.ch (G.D.); rosaria.sacco@eoc.ch (R.S.)

**Keywords:** multiple sclerosis, COVID-19 outcome, SARS-CoV-2 vaccines, SARS-CoV-2 variants, disease-modifying treatment

## Abstract

Patients suffering from neuro-inflammatory diseases such as multiple sclerosis (MS) and neuromyelitis optica spectrum disorders (NMOSD) remain vulnerable to COVID-19. We investigated the risk of COVID-19 in MS and NMOSD patients over time, considering the impact of disease-modifying treatments (DMTs), vaccinations, and the spread of new SARS-CoV-2 variants. We retrospectively collected clinical information regarding all MS and NMOSD consecutive patients seen at the Neurocenter of Southern Switzerland. Logistic regression was used to test variables (age, sex, vaccination status, DMT at vaccination, DMT at infection, disease course, disability scores, prevalent SARS-CoV-2 variant) for association with COVID-19 risk and severe outcome (hospitalization or death). We included 352 individuals in this study; 315 (89.5%) received ≥1 dose of SARS-CoV-2 mRNA-vaccine, and 134 (38.1%) experienced COVID-19 between March 2020 and August 2022. COVID-19 risk decreased in vaccinated patients (OR = 0.10, 95% CI = 0.05–0.20, *p* < 0.001) and increased in anti-CD20 therapies (OR = 2.26, 95% CI = 1.28–4.00, *p* = 0.005). Anti-CD20 treatment was associated with severe COVID-19 (OR = 27.41, 95% CI = 3.68–204.25, *p* = 0.001), whereas Omicron infections were milder compared to Alpha infections (OR = 0.03, 95% CI = 0.01–0.35, *p* = 0.006). We confirmed a protective effect of mRNA vaccines on COVID-19 risk, which is impaired by anti-CD20 treatment. We provided evidence for milder COVID-19 with the Omicron SARS-CoV-2 variant, which should not, however, discourage vaccinations.

## 1. Introduction

During the SARS-CoV-2 pandemic, concerns were raised for fragile and immunosuppressed patients suffering from inflammatory diseases of the central nervous system, such as multiple sclerosis (MS) and neuromyelitis optica spectrum disorders (NMOSD). Despite a death rate related to COVID-19 similar to that of the general population, several studies have shown that the hospitalization rate was higher among patients with MS, and that the risk of severe COVID-19 was augmented in individuals with higher disability scores, greater age or who were treated with anti-CD20 antibodies [1,2,3,4,5].

The introduction of vaccines has been invaluable in preventing severe COVID-19, but disease modifying treatments (DMTs) can influence vaccine efficacy by reducing humoral and cellular responses, thus possibly altering COVID-19 severity [6,7,8,9]. For example, we and others have shown that treatment with anti-CD20 antibodies and sphingosine-1-phosphate receptor (S1P-r) modulators were associated with diminished vaccine-induced SARS-CoV-2 IgG production, as well as SARS-CoV-2 specific memory B cells [10,11]. On the other hand, vaccine-induced T cell responses were blunted in patients treated with S1P-r modulators, but robust among those treated with anti-CD20 [8].

In addition to the development of vaccines, COVID-19 outcomes have also progressively changed, together with the spread of new SARS-CoV-2 variants and the likely repeated exposure to the virus. Monitoring and understanding how COVID-19 outcomes have changed over time, together with repeated vaccinations, infections and the spread of new virus variants, is relevant for the development of appropriate guidelines for COVID-19 prevention, in particular to indicate which patients should be primarily targeted by vaccines. This would also provide important information regarding what can be expected during potential future pandemics.

We therefore aimed to investigate how the risk of COVID-19 and clinical outcomes have changed over the pandemic years in a homogeneous population of patients with neuroinflammatory diseases, simultaneously taking into account the role played by specific DMTs, the development of vaccines and the spread of new SARS-CoV-2 variants.

## 2. Materials and Methods

This was a retrospective single-center observational study conducted at the MS center of the Neurocenter of Southern Switzerland (Lugano, Switzerland). Inclusion criteria were: (1) a diagnosis of MS according to the 2017 revision of McDonald criteria, or neuromyelitis optica spectrum disorders (NMOSD) [12,13]; and (2) agreement to participate in this study. There were no specific exclusion criteria.

All patients consecutively seen at the MS center between March and August 2022 were asked to fill in COVID-19 related questionnaires with the help of the treating neurologist during routine visits. When needed, data collection was completed using medical electronic records, regularly updated at the time of each neurological visit.

The following variables were collected: age, sex, expanded disability status scale (EDSS) score [14], disease course (relapsing vs. progressive), vaccine status, date of vaccination, COVID-19 status (i.e., date and severity of infections), DMT at time of vaccination (if any), DMT at time of infection and treatment of COVID-19 with monoclonal antibodies (i.e., Sotrovimab [15]).

MS course was categorized as either relapsing-remitting MS (RRMS) or progressive MS (PMS, either primary or secondary). DMTs were classified based on their pharmacological properties and mechanisms of action as injectables (interferons, glatiramer acetate), anti-CD20 antibodies (rituximab, ocrelizumab, ofatumumab), other monoclonal antibodies (alemtuzumab, natalizumab, tocilizumab), S1P-r modulators (fingolimod, ozanimod, siponimod) or other oral therapies (dimethyl fumarate, teriflunomide, cladribine, azathioprine, mycophenolate mofetil).

COVID-19 events were all confirmed by a positive PCR nasopharyngeal swab test for SARS-CoV-2. Cases of COVID-19 were defined as severe if the clinical status required hospitalization (including admission to the intensive care unit (ICU)) or led to the death of the patient. Remaining infections were considered mild. These data were retrieved during neurological visits, and by checking electronic medical records of the regional Health Institution (Ente Ospedaliero Cantonale). SARS-CoV-2 variants were inferred based on the predominant circulating variant in Switzerland at the time of infection in each patient (i.e., Alpha for all infections occurring before 25 June 2021, Delta for infections occurring between 26 June 2021 and 21 December 2021 and Omicron for infections occurring after 22 December 2021). These data were collected from the Swiss Federal Office of Public Health (https://www.bag.admin.ch/bag/en/home.html (accessed on 15 March 2023)). This research study was conducted retrospectively using data obtained for clinical purposes, and approved by the Canton Ticino ethics committee.

## 3. Statistics

Categorical variables were described using counts and percentages; continuous and ordinal variables were described using medians and interquartile ranges (IQR). Univariate and multivariate logistic regression models were used to test variables of interest with binary outcomes. First, we tested for association with COVID-19 status (i.e., ever infected vs. not infected) the following independent variables: age (per 1 year increase), sex (female vs. male), MS course (PMS vs. RRMS), EDSS, vaccination status (ever vs. never) and DMTs. Given the evidence for a diminished vaccine response in individuals treated with anti-CD20 and S1P-r modulators, these DMTs were specifically tested against the remaining DMTs or being untreated. Second, the same variables, with the addition of SARS-CoV-2 variants, were tested for association with COVID-19 outcome (severe as compared to mild). All analyses and figures were performed and created using the statistical software R (https://www.r-project.org/ (accessed on 1 December 2021)).

## 4. Results

### 4.1. Patients and Number of COVID-19 Cases

Data from 352 patients were collected between March and August 2022. Of these, 315 patients were vaccinated with mRNA vaccines by August 2022 (Pfizer = 207, Moderna = 95, unknown = 13; 5 with one dose, 67 with two doses, 220 with three doses and 23 with four doses) [16,17]. Vaccinated patients were significantly older than not-vaccinated patients (50.5 [40.8–58.1] vs. 40.3 [32.0–48.3] years, respectively, *p* < 0.001). Baseline demographic characteristics, DMTs at time of vaccination and the number of COVID-19 cases are shown in Table 1.

Overall, 134 patients (38.1%) experienced COVID-19 between March 2020 and data collection. Of these, 10 individuals suffered from two separate SARS-CoV-2 infections (one initial infection and one subsequent re-infection). A total of 315 patients were vaccinated for SARS-CoV-2 within the study period. Of these vaccinated patients, 206 (65.4%) never experienced COVID-19, 82 (26.0%) had breakthrough COVID-19 after vaccination, and 27 (8.6%) were vaccinated after a previous SARS-CoV-2 infection.

**Figure 1:**
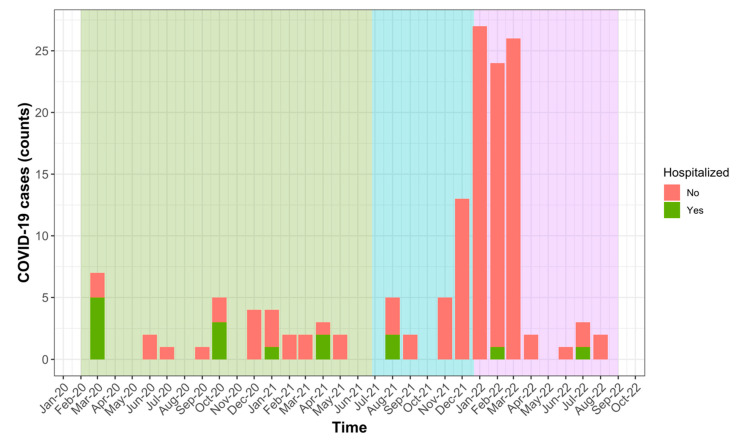
Occurrence of severe (hospitalized) and mild COVID-19 between March 2020 and August 2022 in our population of MS and NMOSD patients. Predominant circulating SARS-CoV-2 variants are shown using background colours: Alpha (green), Delta (blue) and Omicron (violet).

Figure 1 shows the number of COVID-19 cases observed over time among patients included in this study between March 2020 and August 2022. The figure also indicates how many cases required hospitalization and which SARS-CoV-2 variant was prevalent in Switzerland at the time of infection. The largest number of infections occurred during the intervals October 2020–April 2021 (*n* = 20 (13.9%), under Alpha variants) and November 2021–March 2022 (*n* = 96 (66.7%), under Delta and then Omicron variants).

### 4.2. Variables Associated with Risk of COVID-19

We used logistic regression models to test variables for association with occurrence of COVID-19. In univariate logistic regression, greater age (OR = 0.96, 95% CI = 0.95–0.98, *p* < 0.001), higher EDSS (OR = 0.84, 95% CI = 0.74–0.95, *p* = 0.005) and being vaccinated (OR = 0.09, 95% CI = 0.05–0.18, *p* < 0.001) were significantly associated with lower risk of COVID-19. Of these variables, only vaccination status (OR: 0.10, 95% CI = 0.05–0.20, *p* < 0.001) remained significant using the multivariate model, whereas age and EDSS associations were attenuated (*p* = 0.059 and *p* = 0.087, respectively) (Table 2). Interestingly, treatment with anti-CD20 also appeared associated with a higher risk of COVID-19 using the multivariate model (OR = 2.26, 95% CI = 1.28–4.00, *p* = 0.005) (Table 2).

We also investigated variables associated with risk of COVID-19 within the group of vaccinated patients. In the multivariate model, age was inversely associated (OR = 0.98, 95% CI = 0.95–1.00, *p* = 0.042) and anti-CD20 treatment was positively associated (OR = 2.18, 95% CI = 1.18–4.03, *p* = 0.013) with risk of COVID-19 (despite vaccination); the remaining independent variables were not associated with risk of COVID-19.

### 4.3. Variables Associated with Severe COVID-19

Of 144 COVID-19 infected patients, 129 (89.6%) had a mild course and 15 (10.4%) had a severe course requiring hospitalization (Table 3). Among these 15 infections, one severely disabled patient died, and two other patients were admitted to the ICU (one of whom required prolonged intubation). Overall, patients suffering from severe COVID-19 were older compared to patients with mild COVID-19 (50.2 [IQR: 39.2–57.5] vs. 44.3 [IQR: 33.3–51.2] years, respectively) and had higher EDSS scores (3.0 [IQR: 2.1–4.8] vs. 2.0 [IQR: 1.5–3.0], respectively).

**Figure 2:**
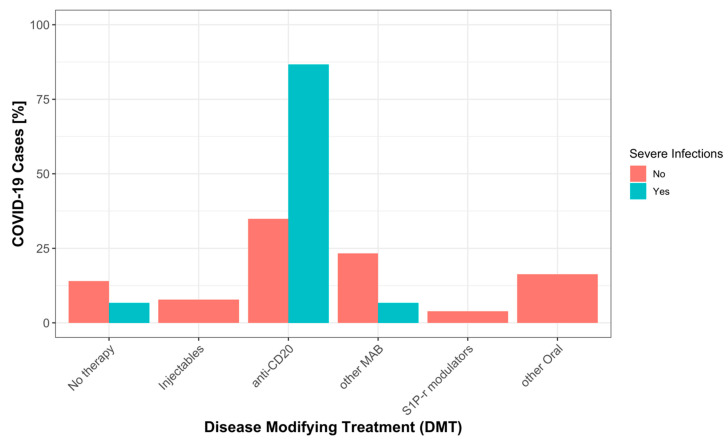
Percentage of severe and mild COVID-19 by DMT categories, as explained in methods: No therapy (no DMT), Injectables (interferons, glatiramer acetate), anti-CD20 (rituximab, ocrelizumab, ofatumumab), other MAB (alemtuzumab, natalizumab, tocilizumab), S1P-r modulators (fingolimod, ozanimod), other Oral (dimethyl fumarate, teriflunomide, cladribine, azathioprine, mycophenolate mofetil).

Notably, 13 of 15 severe COVID-19 cases occurred during treatment with anti-CD20 antibodies (86.7%), one occurred in a patient treated with natalizumab (6.7%), and one patient was under no treatment (6.7%) (Figure 2). Only 26.7% of severe COVID-19 cases occurred in patients who had been vaccinated before infection, as compared to 66.7% of mild COVID-19 cases. There was a high percentage of Alpha variants among severe COVID-19 cases (73.3%), whereas the most frequent variant among mild forms was Omicron (69.8%) (Table 3). The proportion of infections treated with monoclonal antibodies was similar for mild (*n* = 23 (18%)) and severe (*n* = 4 (27%)) COVID-19 cases.

Despite the small number of events, we attempted to investigate variables associated with risk of severe COVID-19 using logistic regression. In univariate models, higher EDSS (OR = 1.37, 95% CI = 1.04–1.82, *p* = 0.028) and treatment with anti-CD20 at time of infection (OR = 11.41, 95% CI = 2.46–52.86, *p* = 0.002) were positively associated with severe COVID-19. In contrast, being vaccinated (OR = 0.18, 95% CI = 0.06–0.60, *p* = 0.005) and infection by the Omicron variant (OR = 0.04, 95% CI = 0.01–0.22, *p* < 0.001) were associated with a lower risk of severe COVID-19. In multivariate analysis, only treatment with anti-CD20 (OR = 27.41, 95% CI = 3.68–204.25, *p* = 0.001) and the Omicron variant (OR = 0.03, 95% CI = 0.01–0.35, *p* = 0.006) maintained a significant association (Supplementary Table S1).

## 5. Discussion

Several factors, including ageing, neurological disability, immunosuppressive therapies and different SARS-CoV-2 variants are likely to influence the response to vaccines and the risk of developing COVID-19. We summarized our experience of COVID-19 in MS and NMOSD patients between March 2020 and August 2022. We investigated how COVID-19 incidence has changed over time, as well as the association between variables of interest with risk of COVID-19 overall and in severe forms, especially in terms of vaccine efficacy and the role played by different SARS-CoV-2 variants.

As for susceptibility to COVID-19, we confirmed that vaccination is associated with a lower risk of infection. Nonetheless, studies assessing the effectiveness of vaccination in people with MS have found SARS-CoV-2 vaccines’ immunogenicity and effectiveness to be similar to that of the general population, with the remarkable exception of individuals on anti-CD20 and S1P-r modulators. In particular, both antibody production and memory B cell responses were impaired when treated with anti-CD20 antibodies. Both humoral and T cell responses were also diminished in patients treated with S1P-r modulators [8,18,19,20]. Accordingly, we found an approximately two-fold increased risk to contract SARS-CoV-2 infection in vaccinated MS patients who were treated with anti-CD20 at time of vaccination. This was especially evident when the effect of vaccination was taken into account in a multivariate model, and within the group of vaccinated individuals. A direct explanation for this originates from several studies showing how individuals being treated with anti-CD20 antibodies have an impaired humoral response to SARS-CoV-2 vaccines [7,21,22,23,24,25]. Lower seroconversion rates have been observed, especially in patients with complete B cell depletion and short time intervals between anti-CD20 infusions and vaccinations [9,10,24]. This is relevant, as lower SARS-CoV-2 titers are directly associated with the risk of breakthrough infections [7,26,27,28].

Additionally, in terms of risk of COVID-19, we found a higher risk of infections in younger and less disabled patients. These associations were significant in univariate analyses, with a trend for significance also in the multivariate model (at least partly influenced by the relatively small sample size of this study). We believe these represent true effects, in agreement with results of other studies showing age and EDSS to be lower in MS patients who contracted COVID-19 [27]. These findings are likely explained by differences in social interactions, behaviours, fear of disease and vaccine hesitancy in patients of different ages and with different disabilities. Indeed, vaccinated patients in our cohort were considerably older than those who were not, similar to patients in the report by Grech et al., who found higher vaccine hesitancy in younger patients with MS and patients with unstable disease [29]. Another study from Italy observed an association between young age at vaccination and risk of COVID-19 infection [30]. Nonetheless, attitudes and behavioural characteristics also play an important role in influencing COVID-19 risk in countries with different social structures, such as Iran, where male gender and employment were identified as the main predictors of COVID-19 incidence [31].

We observed that higher disability scores, treatment with anti-CD20, not being vaccinated and the Alpha SARS-CoV-2 variant were all associated with a higher risk of developing severe COVID-19. Associations were particularly strong for anti-CD20 treatment and the virus variant, which remained the only significant variables in the multivariate model. The association with neurological disability was not surprising, as several studies have reported higher EDSS scores among patients with severe COVID-19 [2,5,32,33]. Similarly, studies have shown that not only breakthrough infections, but also more severe COVID-19, occur more frequently in anti-CD20 treated MS patients [8,26]. We did not find associations between sex and COVID-19 outcome, but our study may have been underpowered in this regard.

The predominant circulating SARS-CoV-2 variant had a major influence on COVID-19 severity. In our cohort, the Omicron variant was significantly associated with a milder disease course compared to the Alpha variant. Notably, this effect appeared to be even stronger than that of vaccination when both factors were analyzed in the same multivariate model. Although the small number of events suggests caution in interpreting the results of the multivariate analysis, it was clear in our cohort that the risk of severe COVID-19 progressively diminished with new circulating SARS-CoV-2 variants. In the general population, the effectiveness of two doses of mRNA vaccination leading to reduced disease severity has been proven for all three predominant variants. However, to achieve the same effectiveness in preventing hospital admissions and lowering disease severity against the SARS-CoV-2 Omicron variant, a third booster dose is needed [34]. The same study found that in unvaccinated patients the Delta variant was associated with more severe COVID-19 as compared to Alpha and Omicron variants [34]. Whether the low risk of severe infections with the latest SARS-CoV-2 variants should change vaccination campaigns for people with MS remains to be determined. We suggest neurologists remain cautious and supportive of vaccination, especially regarding those patients with additional risk factors for severe COVID-19 (i.e., greater disability and treatment with anti-CD20 antibodies).

### Strengths and Limitations

The strengths of this study are represented by the standardized follow-up of the patients at our MS center, with visits performed every 3 to 6 months and regular collection of data regarding vaccination, infection status and treatment history. Furthermore, we consecutively included all patients seen within a specific time interval in this study, which makes the sample well representative of the overall patient population in the region. The main limitation of this study was its retrospective design, which did not permit exclusion of mechanisms of reverse causations behind observed associations and likely multiple confounding factors, for which we attempted to correct using multivariate regression models. Further limitations are represented by the small sample size, exclusion of possible paucisymptomatic untested infections, and the lack of information regarding SARS-CoV-2 antibody titers as a direct measure of vaccine efficacy.

## 6. Conclusions

In conclusion, we confirmed that vaccinations exerted a protective effect on the risk of developing COVID-19 during the period from March 2020 to August 2022 in our patients with neuroinflammatory diseases. Anti-CD20 therapies negatively influenced such protective effect and increased the risk of breakthrough and severe infections. We also provided evidence for a milder disease course with the Omicron (as compared to Alpha) SARS-CoV-2 variant. This should not, however, discourage vaccination among individuals with MS and NMOSD, particularly if they have additional risk factors for severe COVID-19. We believe future studies should maintain monitoring rates and outcomes of COVID-19 in patients suffering from neuro-inflammatory diseases, with the aim of updating appropriate guidelines regarding vaccination and prevention measures.

## Figures and Tables

**Table 1:** Demographic and clinical characteristics of patients included in this study, overall and stratified by occurrence of COVID-19. In this study, vaccination status for individuals who had COVID-19 was considered positive only if patients were vaccinated before infection.

Variables		All Patients	COVID-19	No COVID-19
		(*n* = 352)	(*n* = 134)	(*n* = 218)
Age (years)	Median (IQR)	48.5 (38.9–57.8)	45.1 (33.6–52.6)	51.9 (41.8–60.0)
Sex	F (%)	240 (68.2)	98 (73.1)	142 (65.1)
	M (%)	112 (31.8)	36 (26.9)	76 (34.9)
MS course	RRMS (%)	282 (80.1)	114 (85.1)	168 (77.1)
	SPMS (%)	32 (9.1)	8 (6.0)	24 (11.0)
	PPMS (%)	23 (6.5)	7 (5.2)	16 (7.3)
	NMOSD (%)	15 (4.3)	5 (3.7)	10 (4.6)
EDSS	Median (IQR)	2.5 (2.0–4.0)	2.0 (1.5–3.5)	3.0 (2.0–4.0)
Vaccination status	Yes (%)	315 (89.5)	82 (61.2)	206 (94.5)
	No (%)	37 (10.5)	52 (38.8)	12 (5.5)
DMT at vaccination	No therapy (%)	41 (13.0)		
	Injectables (%)	33 (10.5)		
	Other MAB (%)	61 (19.4)		
	Anti-CD20 (%)	98 (31.1)		
	S1P-r modulators (%)	16 (5.1)		
	Other oral DMTs (%)	66 (21.1)		
Number of COVID-19 infections	0 (%)	218 (61.9)	0 (0.0)	218 (100.0)
	1 (%)	124 (35.2)	124 (92.5)	0 (0.0)
	2 (%)	10 (2.8)	10 (7.5)	0 (0.0)

**Table 2:** Results of univariate and multivariate logistic regression models testing variables for association with occurrence vs. no occurrence of COVID-19. For patients experiencing COVID-19, only vaccinations performed before infections were considered.

Variables		Univariate			Multivariate		
		OR	95% CI	*p*	OR	95% CI	*p*
Age		0.96	(0.95–0.98)	<0.001	0.98	(0.96–1.00)	0.059
EDSS		0.84	(0.74–0.95)	0.005	0.85	(0.70–1.02)	0.087
Sex	M	-	-	-	-	-	-
	F	1.46	(0.91–2.34)	0.119	1.26	(0.73–2.16)	0.403
MS course	RRMS	-	-	-	-	-	-
	PMS	0.56	(0.30–1.06)	0.075	1.01	(0.42–2.41)	0.983
DMTs	Untreated/other DMTs	-	-	-	-	-	-
	Anti-CD20	1.50	(0.94–2.40)	0.091	2.26	(1.28–4.00)	0.005
	S1P-r modulators	1.11	(0.39–3.17)	0.843	1.82	(0.58–5.66)	0.302
SARS-CoV-2 vaccination	No						
	Yes	0.09	(0.05–0.18)	<0.001	0.10	(0.05–0.20)	<0.001

**Table 3:** Demographic and clinical characteristics of patients experiencing mild vs. severe forms of COVID-19.

Variables		Mild COVID-19	Severe COVID-19
		(*n* = 129)	(*n* = 15)
Age (years)	Median (IQR)	44.3 (33.3–51.2)	50.2 (39.2–57.5)
Sex	F (%)	93 (72.1)	12 (80.0)
	M (%)	36 (27.9)	3 (20.0)
MS course	RRMS (%)	112 (86.8)	11 (73.3)
	SPMS (%)	6 (4.7)	2 (13.3)
	PPMS (%)	6 (4.7)	1 (6.7)
	Other (%)	5 (3.9)	1 (6.7)
EDSS	Median (IQR)	2.0 (1.5–3.0)	3.0 (2.1–4.8)
DMT at time of infection	No therapy (%)	18 (14.0)	1 (6.7)
	Injectables (%)	10 (7.8)	0 (0.0)
	Other MAB (%)	30 (23.3)	1 (6.7)
	Anti-CD20 (%)	45 (34.9)	13 (86.7)
	S1P-r modulators (%)	5 (3.9)	0 (0.0)
	Other oral therapies (%)	21 (16.3)	0 (0.0)
Vaccination status at	Yes (%)	86 (66.7)	4 (26.7)
time of infection	No (%)	43 (33.3)	11 (73.3)
Time since vaccination (months)	median (IQR)	3.30 (2.2–5.5)	3.76 (2.9–5.1)
Estimated variant	Alpha (%)	22 (17.1)	11 (73.3)
	Delta (%)	17 (13.2)	2 (13.3)
	Omicron (%)	90 (69.8)	2 (13.3)
Monoclonal antibodies	Yes (%)	23 (18.0)	4 (27.0)
(Sotrovimab)	No (%)	106 (82.0)	11 (73.0)

## Data Availability

Anonymized data not published within this article will be made available by request from any qualified investigator.

## Supplementary Materials

The following supporting information can be downloaded at: https://www.mdpi.com/article/10.3390/jcm12175551/s1, Table S1: Uni- and multivariate logistic regression predicting risk of severe COVID-19 infection leading to hospitalization based on the analysis of all COVID-19 infections (and not individuals). SPMS and PPMS are defined as progressive forms of MS course. Vaccination status is defined as positive for individuals vaccinated before COVID-19 infection. The effect of COVID-19 variants is shown as risk prediction of Delta and Omicron variants in comparison to the first predominant Alpha variant.
