# Supplementary material for: Influence of Disease Modifying Treatment, Severe Acute Respiratory Syndrome Coronavirus 2 Variants and Vaccination on Coronavirus Disease 2019 Risk and Outcome in Multiple Sclerosis and Neuromyelitis Optica
Source: J Clin Med. 2023 Aug 25;12(17):5551. doi: 10.3390/jcm12175551 (PMC10488002; doi:10.3390/jcm12175551)
Supplement: Supplementary file 1 [file jcm-12-05551-s001.zip › jcm-2567945-supplementary.pdf]

SUPPLEMENTARY MATERIALS

Supplementary Table S1. Uni- and multivariate logistic regression predicting risk of severe COVID-19 infection leading to hospitalization based on the analysis of all COVID-19 infections (and not individuals). SPMS and PPMS are defined as progressive forms of MS course. Vaccination status is defined as positive for individuals vaccinated before COVID-19 infection. The effect of the COVID-19 variants is shown as risk prediction of Delta and Omicron variants in comparison to the first predominant Alpha variant.

**Predicting Variables**		**Univariate**			**Multivariate**		
		**OR**	**95% CI**	**p**	**OR**	**95% CI**	**p**
Age		1.04	(1.00 - 1.08)	0.078	1.03	(0.95 - 1.12)	0.481
EDSS		1.37	(1.04 - 1.82)	0.028	1.33	(0.70 - 2.52)	0.382
Sex	M	-	-	-	-	-	-
	F	1.55	(0.41 - 5.81)	0.517	3.09	(0.47 - 20.47)	0.242
MS course	RRMS	-	-	-	-	-	-
	PMS	2.44	(0.60 - 9.86)	0.212	1.2	(0.07 - 19.50)	0.897
DMTs	Untreated / other DMTs	-	-	-	-	-	-
	Anti-CD20	11.41	(2.46 - 52.86)	0.002	27.41	(3.68 - 204.25)	0.001
	S1P-r modulators	0	(0.00 - ∞)	0.994	0	(0.00 - ∞)	0.995
Vaccination	No						
	Yes	0.18	(0.06 - 0.60)	0.005	1.14	(0.13 - 10.06)	0.909
SARS-CoV-2 variant	Alpha variant						
	Delta variant	0.24	(0.05 - 1.21)	0.083	0.19	(0.02 - 2.42)	0.202
	Omicron variant	0.04	(0.01 - 0.22)	<0.001	0.03	(0.00 - 0.35)	0.006
